# Development of functionalized multi-walled carbon-nanotube-based alginate hydrogels for enabling biomimetic technologies

**DOI:** 10.1038/srep32456

**Published:** 2016-08-31

**Authors:** Binata Joddar, Eduardo Garcia, Atzimba Casas, Calvin M. Stewart

**Affiliations:** 1 Department of Metallurgical, Materials and Biomedical Engineering, The University of Texas at El Paso, 500 W University Avenue, El Paso, TX 79968, USA; 2 Border Biomedical Research Center, The University of Texas at El Paso, 500 W University Avenue, El Paso, TX 79968, USA; 3 Department of Mechanical Engineering, The University of Texas at El Paso, 500 W University Avenue, El Paso, TX 79968, USA; 4 Department of Biological Sciences, The University of Texas at El Paso, 500 W University Avenue, El Paso, TX 79968, USA

## Abstract

Alginate is a hydrogel commonly used for cell culture by ionically crosslinking in the presence of divalent Ca^2+^ ions. However these alginate gels are mechanically unstable, not permitting their use as scaffolds to engineer robust biological bone, breast, cardiac or tumor tissues. This issue can be addressed via encapsulation of multi-walled carbon nanotubes (MWCNT) serving as a reinforcing phase while being dispersed in a continuous phase of alginate. We hypothesized that adding functionalized MWCNT to alginate, would yield composite gels with distinctively different mechanical, physical and biological characteristics in comparison to alginate alone. Resultant MWCNT-alginate gels were porous, and showed significantly less degradation after 14 days compared to alginate alone. *In vitro* cell-studies showed enhanced HeLa cell adhesion and proliferation on the MWCNT-alginate compared to alginate. The extent of cell proliferation was greater when cultured atop 1 and 3 mg/ml MWCNT-alginate; although all MWCNT-alginates lead to enhanced cell cluster formation compared to alginate alone. Among all the MWCNT-alginates, the 1 mg/ml gels showed significantly greater stiffness compared to all other cases. These results provide an important basis for the development of the MWCNT-alginates as novel substrates for cell culture applications, cell therapy and tissue engineering.

Hydrogels are popularly used as two- and three-dimensional culture scaffolds for cells because they closely mimic cells’ natural physical and chemical environments^1^,^2^. In addition, hydrogels are extremely biocompatible and, biodegradable, and they can be used to encapsulate growth factors and cells to engineer tissues in a dish *in vitro*^2^. They can be fabricated from synthetic (e.g., poly(ethylene glycol), poly(hydroxyethyl methacrylate)) or from naturally occurring polymers (e.g., collagen, hyaluronan, heparin and alginate). They also have high water content and the ability to crosslink in the presence of divalent cations^2^. Cells^3^, proteins^4^ and DNA^5^ can be incorporated into these hydrogels prior to gelation thus making them applicable to a wide variety of fields in biomedicine. Depending on the reactivity of the constituents, gelation can be induced using pH, temperature variants and other chemical interactions^2^. Alginate or alginic acid is an anionic polysaccharide which crosslinks ionically in the presence of divalent calcium ions and is extensively used for making alginate gels for cell culture^3^,^4^,^6^. Alginate gels have been used in applications ranging from drug delivery to cell encapsulation and are usually produced by dripping alginate solution into a CaCl_2_ bath^3^,^7^. The major disadvantages are that the gelation rate is hard to control, so the resulting structure is neither uniform; nor mechanically strong, and complex-shaped 3D structures are difficult to achieve^7^,^8^. Further this leads to uncontrollable degradation, poor cell adhesion and infiltration^1^. Therefore attempts have been made to enhance the mechanical properties of alginates by reinforcing a secondary phase, such as carbon nanotubes (CNT)^6^,^9^.

CNT have varying properties that make them appealing for various applications ranging from electronics to biomedicine^10^,^11^. They can be single- (SWCNT) or multi-walled (MWCNT) which are usually non-functionalized in their pristine form. Non-functionalized CNT need to be functionalized via introduction of carboxyl (-COOH) or hydroxyl (-OH) groups to make them soluble in aqueous medium^12^. The hollow structure and high aspect ratio of CNT affords great mechanical strength and electrical properties^10^,^11^. These properties have allowed CNT to be applied as reinforcing phases in tissue engineering to fabricate robust scaffolds for tissue and cell proliferation^6^,^13^,^14^,^15^,^16^. While these CNT-hydrogels can serve as efficient substrates for drug immobilization and sustained release^17^, the mechanical properties of these CNT-based composite gels have not been reported^6^, or correlated with the biological behavior of cells cultured atop such substrates^6^.

In this study, composite MWCNT-alginates were developed by encapsulating COOH-functionalized MWCNT as the reinforcing phase within alginate. The concentration of MWCNT encapsulated within alginate was varied to investigate the effects on resultant mechanical and other physical properties. Furthermore, the biocompatibility of these composite gels was evaluated using HeLa-GFP^18^. Results will help establish MWCNT-alginates as a new substrate for mimicking cancer progression in a dish, or for cell therapy and tissue engineering. To our knowledge, this is the first time functionalized MWCNT-based alginates have been fabricated and tested for both mechanical properties and biocompatibility. It is also the first time causal relationships between these properties have been established.

## Results

### Development and characterization of MWCNT-alginate gels

**Figure 1:**
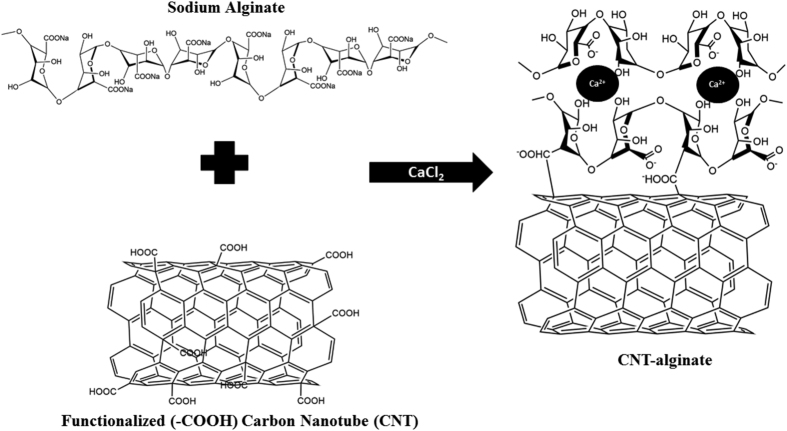
Chemical scheme for CNT-alginate formation. The CNT binds to alginate via covalent bonding.

**Figure 2:**
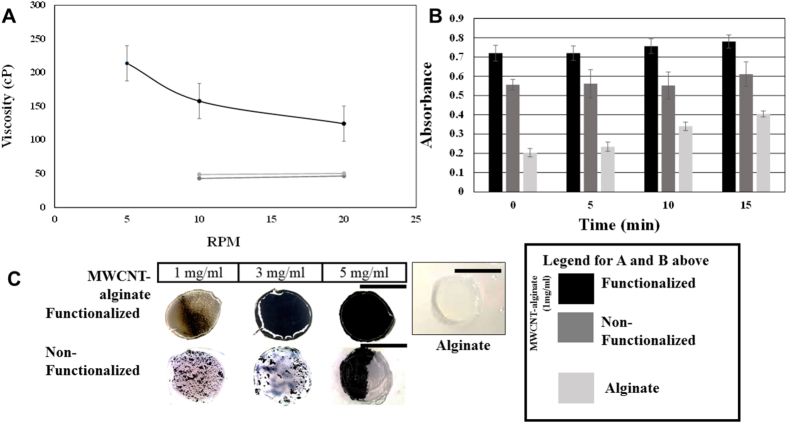
(**A**) Viscosities of MWCNT-alginate (1 mg/ml) plotted versus increasing shear rates (rpm). (**B**) Absorbance assay tracking gel formation for MWCNT-alginate (1 mg/ml) at increasing time points from 0–15 min. For A and B, legend in shown in box in the bottom right corner. (**C**) Representative e*n-face* images of MWCNT-alginate gels both functionalized (top) and non-functionalized (bottom) shown for comparison. Scale bar is 4 mm in the top panel and 2 mm in the bottom panel respectively. Shown also is a representative disc-shaped punch out gel of pure alginate (scale bar = 4 mm).

Functionalized MWCNT (with -COOH groups) were homogenized and incorporated within alginate (Fig. 1). Non-functionalized pristine MWCNT were used as controls to show the comparison among the dispersion characteristics of both functionalized and non-functionalized MWCNT^6^ in alginate. The alginate and the non-functionalized MWCNT-alginate solutions behaved as Newtonian fluids; their viscosities did not change as a function of increasing shear rate at a constant temperature of 25 °C (Fig. 2A). However, both these fluids were plastic since they did not start flowing until sheared at 10 rpm. In contrast, the functionalized MWCNT-alginate solutions exhibited a typical Non-Newtonian pseudoplastic or shear-thinning behavior characterized by decreasing viscosities with an increasing shear rate, as shown in the Fig. 2A. These non-functionalized MWCNT-alginate solutions (1 mg/ml) retained constant viscosities even at increasing shear rates, because the MWCNT formed cohesive clusters which did not disperse throughout the alginate during shearing and did not account for change in viscosity compared to the alginate alone. Uniform sized gels were cast following procedures outlined in Supplementary Figure 1 and videos at https://www.youtube.com/watch?v=G4PM4C3_vnA&feature=youtu.be and at https://www.youtube.com/watch?v=QaYjWQbGw0I.

Absorbance measurements of the viscous MWCNT-alginate solutions were necessary to ensure that incorporation of MWCNT would not hinder gelation of alginate. An absorbance assay was performed in order to confirm the gelation of the MWCNT-alginate solution after addition of calcium chloride (CaCl_2_). MWCNT-alginate solutions showed increasing absorbance intensity confirming gel formation. Beyond 15 min., the absorbance intensity did not change confirming the end point of gelation (Fig. 2B). However, the MWCNT-alginates, both functionalized and non-functionalized, required longer time to gel compared to alginate alone (Fig. 2B). The functionalized MWCNT-alginate gels looked well-homogenized in comparison to the non-functionalized gels that showed distinct areas of encapsulation with and without MWCNT (Fig. 2C). From this point onward, all experiments were done using functionalized MWCNT since the objective was to uniformly disperse the MWCNT throughout the alginate gels, which was possible only by employing functionalized, and not non-functionalized, MWCNT.

Functionalized and non-functionalized MWCNT could also be incorporated within collagen and form gels showing the applicability of MWCNT as a reinforcing phase for other hydrogels (Supplementary Figure 2). However only ~10% v/v of MWCNT could be incorporated within the collagen gels, otherwise hindering gelation of the collagen (37 °C for 1 hr.).

**Figure 3:**
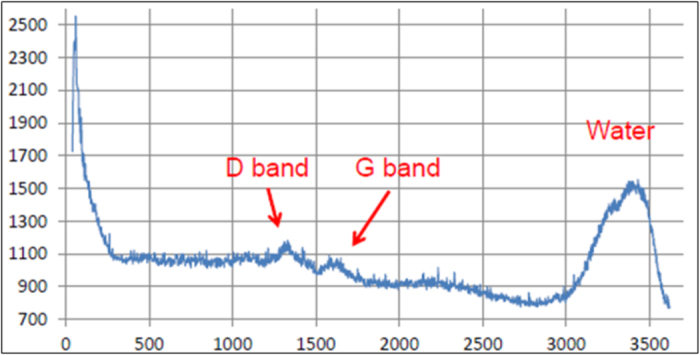
Representative Raman-spectra of a MWCNT-alginate (1 mg/ml) gel sample showing the characteristic bands in MWCNT.

MWCNT-alginate gels (1 mg/ml) were analyzed with AFM/Raman spectra to confirm the presence of encapsulated MWCNT (Fig. 3). Characteristic D-band and G-bands, as well as bands corresponding to water in the Raman spectra, were obtained from the MWCNT-alginate (1 mg/ml) which confirmed the incorporation of MWCNT within alginate after gelation (Fig. 3)^19^.

**Figure 4:**
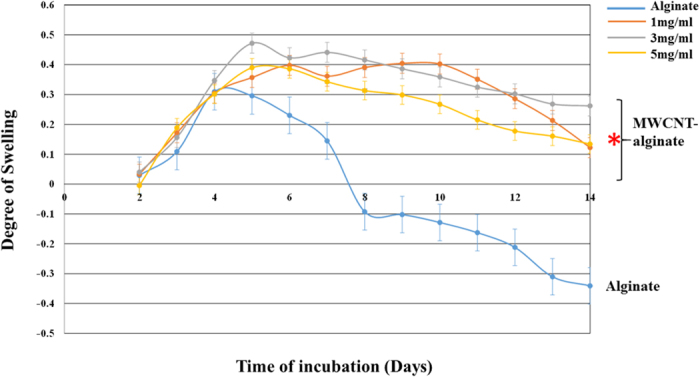
Swelling and degrading of MWCNT-alginate (1, 3, 5 mg/ml) tracked during 14 days of incubation in DMEM. The MWCNT-alginate gels showed significantly less degradation compared to alginate alone (*).

The swelling kinetics and behavior of MWCNT-alginate gels in Dulbecco’s Modified Eagle’s Medium (DMEM) are plotted in Fig. 4. There were significant differences among the swelling and degradation behavior of MWCNT-alginate and alginate only (Fig. 4). The MWCNT-alginate gels had comparatively higher swelling ratio, while alginate gels had the lowest. However, the MWCNT-alginate gels took longer time (~6 days) to reach their equilibrium swelling, compared to alginate which reached this limit after 4 days of incubation. Beyond this time point, alginate gels started degrading and became mechanically very fragile. For the MWCNT-alginates, the swelling ratios remained relatively unchanged throughout the remaining incubation period even after their equilibrium swelling point was reached. Thus, by incorporating MWCNT within alginate, the overall stability of the gels was increased. But, since the MWCNT were not extensively crosslinked to the alginate, more water was absorbed within the gel leading to higher swelling ratios compared to alginate. This phenomenon can be better controlled if approaches to better crosslink the MWCNT with the alginate are implemented.

**Figure 5:**
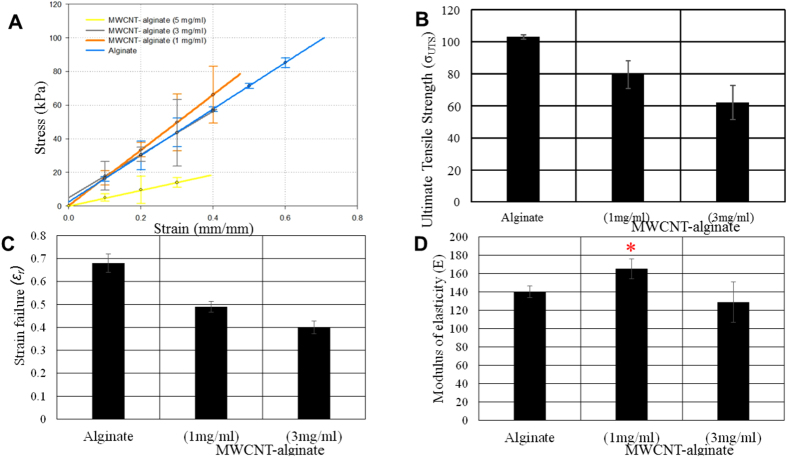
(**A**) Stress-strain curves of functionalized MWCNT-alginate (1, 3, 5 mg/ml) and controls. (**B**) Ultimate stress, (**C**) Strain Failure and (**D**) Elastic Moduli (**E**) values for MWCNT-alginate (1 and 3 mg/ml) and controls. Modulus of elasticity for MWCNT-alginate (1 mg/ml) was significantly higher compared to other groups in (**D**).

Monotonic tensile tests were performed on the functionalized MWCNT-alginate gels (1, 3, 5 mg/ml), and pure alginate hydrogels. Stress versus strain graphs were filtered and plotted together for the alginate and MWCNT-alginate hydrogels. The Modulus of Elasticity, (E), was calculated from the slope of the linear filtered stress-strain curves calibrated from the data points obtained (Fig. 5 and Table 1 below). The average rupture strain, (*ε*_*r*_), ultimate tensile strength, (*σ*_UTS_), and modulus of elasticity, (E), for the 1 mg/ml MWCNT-alginate samples were 0.492 ± 0.02, 79.5 ± 8.5 kPa and 165.2 ± 10.8 kPa respectively in comparison to the alginate gel controls which had *ε*_*r*_ of 0.6434 ± 0.04, *σ*_UTS_ of 103.5 ± 1.4 kPa and E of 140 ± 6.4 kPa respectively. These properties were also evaluated for the 3 mg/ml MWCNT-alginate, which revealed lower values for all of these parameters compared to the 1 mg/ml MWCNT-alginate samples due to the increasing pore size of the former (*ε*_*r*_ = 0.405 ± 0.03, *σ*_UTS_ = 62 ± 11 kPa, and E = 128.9 ± 22 kPa). The results also showed that while the strain to failure and ultimate tensile strength of the alginate decreased with increasing MWCNT concentration, the modulus of elasticity exhibited a significant increase in stiffness around 1 mg/ml in MWCNT-alginate but not in the other MWCNT-alginate gels. This was contradictory to our central hypothesis as we had expected to fabricate gels of increasing stiffness simply by adding increasing amounts of MWCNT to alginate. However by encapsulating only 1 mg/ml MWCNT within alginate gels, stiffer hydrogels could be fabricated (p = 0.037) which are comparable to the values reported for breast tumors tissue by other studies (164.3–169.7 kPa)^20^. But these samples had lower ultimate tensile strength compared to alginate alone. This could be because the MWCNT were weakly chemically bonded to the alginate. Possibly the method used in this study was not enough to overcome the van der Waals forces existing between the MWCNT to disperse them evenly and permit enhanced binding with the alginate. Others have used ultrasound-based methods of dispersion to overcome these existent van der Waals forces between the MWCNT to enable their homogenous distribution throughout gels^21^. The 5 mg/ml MWCNT-alginates were significantly weaker compared to all other samples tested (*ε*_*r*_ ~ 0.3382, *σ*_UTS_ ~ 18 kPa, and E ~ 34.643 kPa).

**Figure 6:**
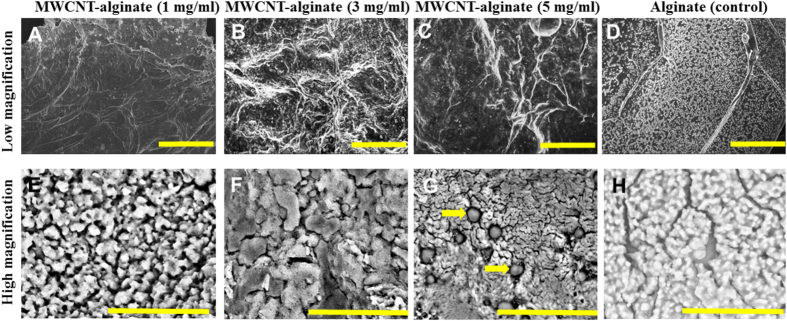
Gel ultrastructure obtained by SEM of MWCNT-alginate (1, 3, 5 mg/ml) and controls. Scale bar in the upper panel is 100 μm and in the lower panel is 20 μm. In G the arrows point to MWCNT agglomerated within the alginate.

Evaluation of gel microstructure and pore-size was performed using scanning electron microscopy (SEM). The SEM micrographs (Fig. 6) showed significant differences in the underlying ultrastructure of the MWCNT-alginate gels compared to alginate controls. *En-face* sections showed textured surfaces in gels with MWCNT encapsulated, unlike alginate controls which appeared smooth on the surface (Fig. 6A–D). This observation was similar to other’s published SEM images^6^. Although alginate gels were porous, they showed evidence of a weaker structure due to the presence of multiple cracks (Fig. 6H). Pore sizes proportionately increased with increasing concentration of MWCNT as resultant images revealed an average pore size of 1.98 ± 1.08 μm (1 mg/ml of MWCNT-gel), 1.96 ± 0.82 μm (3 mg/ml of MWCNT-gel) and 2.22 ± 1.08 μm (5 mg/ml of MWCNT-gel) in comparison with alginate that showed an average pore size of 1.01 ± 0.39 μm (Table 1). However, with increasing concentration of MWCNT, the gels became more brittle as evidenced by the cracks in the structures (Fig. 6B,C), and pores appeared to be non-homogenously distributed and non-interconnected. The average pore size was much higher in the 5 mg/ml MWCNT-alginates because of the evidence of multiple crevices, along with evidence of the MWCNT coagulating into a separate phase in comparison with the other cases. The cross sections of the gels revealed laminated sheets closely connected in a layer-by-layer confirmation (Supplementary Figure 3). The presence of pores in hydrogels plays a critical role in guiding neo-tissue formation and function as it allows homogeneous cell distribution and facilitates interconnection throughout neo-engineered tissues^22^. A future study will explore whether pore sizes can be reduced without affecting mechanical integrity by using SWCNT instead of MWCNT. All the MWCNT-alginates showed evidence of interconnected pores, and the average pore size was significantly greater than control alginate gels (p = 0.000). However, increased pore size related to overall porosity (MWCNT-alginate 5 mg/ml) can also compromise the mechanical properties of a scaffold. Therefore a balance needs to be maintained between the structure and function of porous scaffolds for optimizing mechanical and biological performance^23^. From SEM analysis, it is clear that alginate by itself forms a brittle structure, which is not evident when the gels are encapsulated with MWCNT (1 mg/ml). However, when the concentration of MWCNT exceeds a critical limit beyond 3 mg/ml, this benefit is lost and the structures appeared brittle again.

### Biocompatibility of the MWCNT-alginate gels

**Figure 7:**
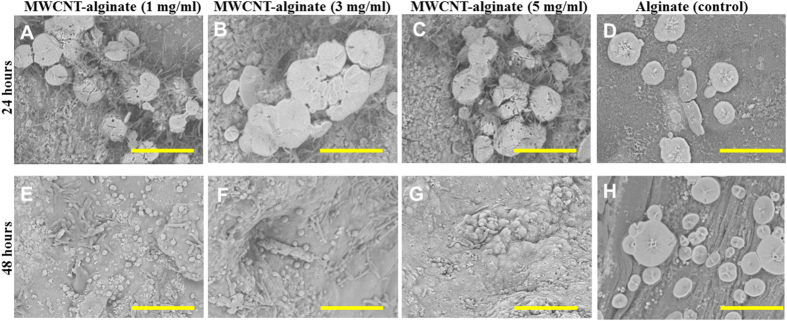
HeLa cultured atop MWCNT-alginate (1, 3, 5 mg/ml) and controls captured using SEM after 24- and 48 hr. respectively. After 24 hr. cells formed clusters on all MWCNT-alginate gels (**A–C**), but not on alginate (**D**). After 48 hr. the formed cell clusters appeared to have migrated throughout the pores of the MWCNT-alginates towards the interior, hence were not detected on the surface (**E–G**). However for the alginate gels, cells were still detected atop the surface of these gels (**H**). Scale bar is 50 μm in all images shown.

To determine the overall biocompatibility and to explore the effects of increasing concentration of MWCNT within alginate, HeLa cells were cultured atop these substrates. From other published studies using other cell types, it is already known that MWCNT-alginates are non-cytotoxic and promote cell adhesion and clustering^6^. In line with these published observations, it was seen that HeLa cells adhered to all gels and controls and appeared viable throughout culture (Supplementary Figures 4 and 5). Further, in the wells with gels, cells adhered preferentially to the gels, and not to the well surface after 24 hours of seeding. As the MWCNT concentration was increased, cell cluster formation was enhanced (Supplementary Figures 4 and 5), which was further confirmed using SEM (Fig. 7). In addition, cells seemed to cluster via secretion of extracellular matrix (ECM) proteins (Fig. 7A–C) atop the MWCNT-alginates in comparison with alginate only (Fig. 7D). After 48 hours of culture, no cells were seen on the surfaces of the MWCNT-alginates (Fig. 7E–G) in comparison with alginate atop which cells were still visible (Fig. 7H). However, a lot of ECM proteins were found deposited on the MWCNT-alginates (Fig. 7E–G). Apparently, the cells had migrated towards the interior of the MWCNT-alginate gels via pores present in the MWCNT-alginates, and so they could not be detected on the surface although they could be visualized using confocal microscopy (Supplementary Figures 4 and 5). Thus, the MWCNT-alginates permitted cell migration in comparison to alginate alone. The SEM morphology of the HeLa were similar to other’s published images^24^.

There is little doubt that cluster formation of HeLa was induced by MWCNT present in the alginate; however, the mechanism via which cell-clustering is induced is not yet clear. Many possible explanations account for cell clustering, and one would be the presence of pores in the scaffold (Fig. 6 and Table 1). Pores in a 3D scaffold can allow cell migration and cross-talk permitting enhanced synthesis of ECM proteins^25^, which in turn can lead to cell aggregation and cluster induction^26^ (Fig. 7A–C). Further, these ECM proteins can reinforce and strengthen the scaffold. Another explanation might be that cell clustering is induced by increasing stiffness of the scaffold (Fig. 5D), which leads to enhanced cell clustering observed in 1 mg/ml MWCNT-alginate gels. Others have stated that cell clustering can also be induced by a charge-effect, due to the presence of free carboxyl groups in the functionalized MWCNT^27^. So, with an increased dose of MWCNT (3 mg/ml) encapsulated, the clustering effect of cells was enhanced.

These cell clusters which were abundantly noted after 24 hours of culture atop MWCNT-alginate gels (Fig. 7A–C) but not on alginate (Fig. 7D), seemed to have migrated within the gels after 48 hours in the MWCNT-alginate gels leaving behind an abundance of ECM protein deposition on the surface (Fig. 7E–G). However cells were still retained on the surface of the alginate gels after 48 hours (Fig. 7H). These results implied that the MWCNT-alginate gels not only promoted cell clustering but also cell migration compared to alginate, which is an important aspect to capture in studies mimicking cancer-on-a-dish^28^.

**Figure 8:**
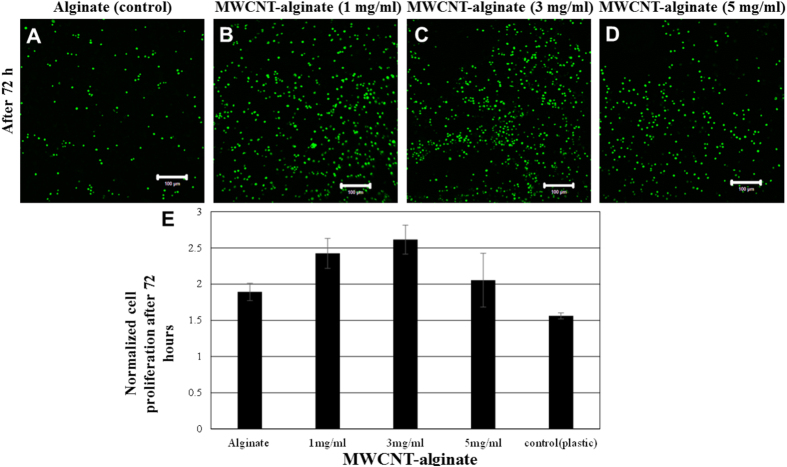
Representative images of HeLa-GFP cells clustering atop MWCNT-alginate (1, 3, 5 mg/ml) (**B**–**D**; Supplementary Figures 4 and 5) and controls (alginate: **A**; plastic: Supplementary Figure 6) along with a quantification of cell proliferation depicted in E after 72 hours of culture.

Visually, cells proliferated on all MWCNT-alginates and controls (Supplementary Figures 4, 5 and 6) compared to alginate only which showed an apparent reduction in viable cell density across the scaffold beyond 48 hours of culture (Supplementary Figure 4H). HeLa cultured on MWCNT-alginates showed signs of proliferation from an early time point of 24 hours, therefore these substrates could not be cytotoxic for cell culture (Supplementary Figure 5). Cells proliferated more on the gels compared to plastic wells, as the gels offered more area for cell adhesion and growth (Fig. 8). Among the gels, cells proliferated to a greater extent on the MWCNT-alginates compared to alginate only (Fig. 8), with a maximum amount of cell proliferation observed on the MWCNT-alginate 3 mg/ml gels (Fig. 8E). The normalized cell proliferation values on 3 mg/ml MWCNT-alginate were significantly greater compared to other gels including alginate (p = 0.011666903) and 5 mg/ml MWCNT-alginate (0.006582316); but not significantly different from 1 mg/ml MWCNT-alginate (p > 0.05). It is not well understood why the extent of cell proliferation was apparently reduced atop 5 mg/ml MWCNT-alginate, but it could be attributed to the presence of increased number of pores and crevices in these gels via which cells could have leaked out from the gels into the tissue culture wells throughout the entire culture period.

## Discussion

The mechanical stiffness of the culture substrate influences many cellular traits including differentiation, adhesion, migration and proliferation^29^,^30^,^31^. There is convincing evidence that cancer progression is driven in part by stiffening of the basal matrix over time, and the stiffness of the tumor generally increases with advancement of the disease^32^,^33^,^34^. In addition, stiffer substrates are also required to mimic scaffold properties to engineer cardiac^13^ and bone^35^ tissues. By homogenously incorporating MWCNT within alginate gels, we expected to fabricate scaffolds of enhanced mechanical properties and simultaneously address the inherent drawbacks of alginate hydrogels while maintaining their high porosity, biocompatibility and biodegradability. This is an important step prior to extensive use of CNT-based hydrogels for biomedical applications^11^. Carbon-nanotube-based scaffolds have been used to regenerate bone^35^ and nerves^36^ and to culture stem cells^37^. In recent years, a large variety of nanomaterials, including carbon-based, polymeric, ceramic and metals have been encapsulated within hydrogels to obtain composite hydrogels with superior properties and desired functionality^38^. Nano-composite hydrogels can be engineered in this manner with advanced physical, chemical, electrical and biological properties^38^. Since alginate is very popular for tissue engineering and cell culture applications^1^,^7^,^39^, attempts have been made to reinforce their mechanical properties using nanomaterials such as CNT^6^. However, to our knowledge this is the first attempt to fabricate uniform sized functionalized MWCNT-alginate hydrogels using a simple solution casting assembly method. Further, we had to encapsulate much lower doses (<10 mg/ml) of MWCNT as we used functionalized MWCNT, compared to a much higher dosage of non-functionalized single-walled CNT reportedly used by others^6^.

The goal was to fabricate hydrogels of varying stiffness from MWCNT and alginate by varying the amount of MWCNT encapsulated within the alginate. Although the modulus of elasticity of alginate was significantly enhanced by adding 1 mg/ml of MWCNT, this failed to increase the ultimate tensile strength of these samples since the MWCNT used were only 8% carboxylic acid functionalized^40^. If the extent of functionalization in the MWCNT is increased (>8%), the bonding strength between the MWCNT and alginate matrix can be increased to yield more mechanically rigid hydrogels. Further, the Ca^2+^ concentration can be increased to yield stronger gels. Also the stress gradients due to clustering of the MWCNT within alginate gels can be reduced by aligning the CNT within alginate as a reinforcing scaffold structure^6^,^9^, ultimately yielding a stronger scaffold. Better alignment of the CNT within alginate can be achieved by inkjet printing^6^,^41^. Others have reported that the use of UV irradiation^42^ enhances chemical crosslinking of the CNT and interaction with the matrix phase, which can be an option to increase the mechanical rigidity and integrity of the composite structure. Hydrogels are often utilized as cell transplantation vehicles, and so their degradation behavior can in turn affect their mechanical properties which are critical factors in new tissue formation. If the prepared gel degrades in an accelerated manner, it will in turn deteriorate their function as a mechanical support in parallel^43^.

Incubation of hydrogels in culture medium and resultant ageing is known to affect their mechanical integrity and degradation^44^. The effects of ageing on mechanical properties^44^ have not been explored. Nonetheless, the gels could maintain their integrity and resist breakdown due to culture for at least 14 days of culture. Further, it is implied that adding CNT to alginate improves the viscoelastic properties of the composite gels formed. By adding MWCNT to alginate, the resultant gels behaved in a ‘sticky’ manner which probably enhanced initial cell adhesion onto these substrates. This resulted in cell clustering, proliferation and migration without the inclusion of RGD-based peptides in the scaffolds^45^.

Among the MWCNT-alginate gels, although the 3 mg/ml gels showed the maximum amount of cell clustering and proliferation, based on an assessment of swelling and mechanical properties, the 1 mg/ml gels were the most rigid and stable. Therefore, approaches to optimize the biological and mechanical properties of these gels need to be performed, and concentrations ranging from ≤1–3 mg/ml MWCNT-alginate also need to be investigated. Further, methods such as increasing the extent of functionalization of CNT^12^,^46^ to enhance chemical crosslinking between the MWCNT and alginate need to be implemented. Nonetheless, by incorporating MWCNT within alginate, HeLa cell cluster formation was significantly enhanced, which makes this substrate particularly appropriate for mimicking cancer progression *in vitro*. Besides, 3D hydrogels provide more surface area for cells to adhere, migrate and proliferate because of the presence of pores which in turn induces cellular morphological changes due to ECM protein secretion^26^. These MWCNT-alginates can be used to study pancreatic^47^, breast^48^, and other types of cancer cells and can be applied to culture colony forming stem cells as well^49^. The concept of embedding nano-/particles within a matrix is not novel as others have incorporated nano-beads within alginate to create porous structures^50^. However, the MWCNT-alginates appeared to have interconnected pores and are seemingly more attractive for cell culture. Ramamurthi *et al*.^51^ used UV to create pores and crevices within hyaluronan hydrogels whereas by incorporating MWCNT within alginate, the pore sizes of the resultant gels were enhanced without any further additional procedures.

HeLa cells showed preferential migration, adhesion onto the gels, especially on the MWCNT-alginates, compared to the alginate alone. This in turn also enhanced cellular ECM production which served to reinforce the scaffold mechanical properties and improved handling characteristics of the MWCNT-alginates, compared to alginate gels with advancing culture time. One drawback of the MWCNT-alginates is that compared to flat well surfaces, comparatively a greater cell seeding density is needed to observe cluster formation. On the other hand, cancer cell migration can be observed in 3D on the gels, but not on the flat surfaces which is extremely important for correlation of ECM matrix stiffness to cell migration in tumor physiology^52^. The encapsulation of MWCNT can be further extended to other gel types both natural and synthetic, such as in collagen and probably to PEG also.

In conclusion the benefits of this particular functionalized MWCNT-alginate scaffolds (1–3 mg/ml) are better handling characteristics and stability, improved stiffness, allowing enhanced cell clustering, ECM production and migration all of which together make these composite gels a better substrate for mimicking cancer progression *in vitro* compared to alginate alone.

## Methods

### Experimental

MWCNT both non-functionalized (pristine) and functionalized with –COOH groups and anhydrous calcium chloride (CaCl_2_) were obtained from Sigma-Aldrich, MO, USA. Phosphate buffered saline (PBS) buffer solution (1X) and Dulbecco’s Modified Eagle Medium (DMEM) was obtained from Gibco, Invitrogen, CA. Alginic Acid was acquired from Acros Organics (NJ, USA). Fetal calf serum was acquired from Hyclone Laboratories (UT, USA). 3D collagen culture kit was procured from EMD Millipore (MA, USA) and used as per manufacturer’s instructions^53^.

MWCNT in their pristine form were insoluble in aqueous media and in alginate; thus, they needed to be functionalized to solubilize and disperse them in aqueous solutions. MWCNT were incorporated in alginate by homogenization (40 min., 25 °C) in a sonicator (Branson B2510MTH, NY, USA) prior to gelation.

### Viscosity and absorbance measurements

To distinguish between functionalized and non-functionalized MWCNT, both types were encapsulated into alginate gels respectively. One set of control gels had non-functionalized MWCNT dispersed within alginate random mixing and sonication prior to gelation. The other set of controls were alginates only. Viscosity measurements were performed and gel formation was tracked using absorbance measurements after incorporating MWCNT within alginate.

Rheological studies of MWCNT-alginate solutions containing 1 mg/ml of functionalized or non-functionalized MWCNT at 25 °C were done prior to gel formation (Rheometer Brookfield cone-plate model LV-DVIII, MA, USA)^54^. Absorbance measurements were done to track gel formation as a function of time (ELx800 Absorbance Reader, BioTek, Winooski, VT, USA). Premixed solutions were added to a 96 well plate and placed on a shaker prior to the addition of CaCl_2_. After CaCl_2_ was added to the wells with sterile pipette tips, gelation was initiated. Absorbance was read at 450 nm prior to gel formation (0-min), as well as after the addition of CaCl_2_ to MWCNT(1 mg/ml)-alginate after 5, 10, and 15 minutes respectively^55^. Controls included solutions of alginate and CaCl_2_ only.

To check the versatility and applicability of MWCNT as a reinforcing phase, it was also incorporated into collagen and formed into gels^55^. Digital images of MWCNT-alginates and MWCNT-collagen gels were acquired using an upright Leica M205C microscope (Leica Microsystems, Buffalo Grove, IL, USA).

### Fabrication of uniform sized MWCNT- alginate gels

Alginate gels of uniform thickness were made following published procedures^56^ using a casting system as described. A 2% (w/v) alginate solution was made by adding alginic acid to 1X PBS buffer, then autoclaved to ensure dissolution and sterilization before sterile-filtration with a 40 μm Nylon Mesh (Fisher Scientific). The sterile alginate solution was then pipetted into a cast composed of two glass slides (non-tissue culture treated) separated by 3D printed spacers printed using a Desktop Replicator (Makerbot Industries, NY, USA) of 1.0 mm thickness throughout the length of the sample. This assembly was held in place by paraffin film wrap, and the entire assembly was submerged into a 0.25 M CaCl_2_ bath (48 h, 25 °C) for gelation to be completed and formed gels to be stabilized (Supplementary Figure 1). To create various doses of MWCNT-alginate gels, different amounts (1 mg/ml, 3 mg/ml, 5 mg/ml) of non-functionalized and functionalized MWCNT were incorporated within alginate and cast into gels.

### Atomic Force Microscopy (AFM)/Raman Spectroscopy

To ensure the incorporation of MWCNT within alginate gels, Raman spectra of these MWCNT-alginates were recorded. After gelation was complete and gels (MWCNT-alginate 1 mg/ml) casted, they were prepared for AFM/Raman spectra analysis (NT-MDT America, Tempe, AZ). NT-MDT scanning probe microscope NTEGRA was used and samples were kept hydrated during this procedure^57^. The Raman spectra were acquired with an AFM/Raman setup NTEGRA Spectra (NT-MDT) and DXR Raman microscope (Thermo Fisher Scientific) configured with 633 nm laser. Raman spectra were obtained using a 100X objective, 50 μm aperture of the confocal module and the laser power at the sample was maintained at 2 mW to obtain high quality Raman spectra and to avoid overheating of the samples^58^.

### Swelling behavior

To account for the hydration parameters of the MWCNT-alginate gels leading to swelling, gels were allowed to swell to equilibrium for 14 days in DMEM (pH = 7, 25 °C) as reported by others^59^,^60^,^61^. This was done to identify the time point when the weight of the gels was found to be constant, or the final swelling degree was attained. Disc-shaped punch out samples (MWCNT-alginate 1, 3, 5 mg/ml respectively and control alginate; each case in triplicate) of surface area ~0.95 cm^2^ were surface-dried and excess liquid was blotted off using blotting paper (Kimwipes, Kimberly Clark, USA) prior to being exposed to the DMEM (W_0_). The gels were then allowed to swell during which they were taken out at regular intervals of 1 day, excess surface liquid was absorbed using blotting paper and the gels were weighed (W_t_). The swelling ratio was calculated using the following equation (1),


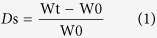


where D_s_ was the degree of swelling, W_0_ and W_t_ were the weights of the samples in the dry and swollen states respectively^62^.

### Mechanical testing

All mechanical tests were done using an ElectroForce 5100 Biodynamics Test Instrument from ElectroForce Systems group (Bose Corporation, Framingham, MA). The machine is equipped with a 200 N (45 lb) load cell with a total of 12 mm of cross-head displacement and can run various precision tests for dynamic materials characterization^63^. For testing, a dog-bone sample was cut using a mold on the MWCNT-alginate hydrogels and carefully mounted between pressure grips. The axis of the specimens was aligned with the center of the grips for uniform axial stress, and the pressure grips were evenly tightened to prevent slippage without crushing the sample^63^. Mounted specimens had an estimated cross-sectional area of 5 mm and a gauge length of 15 mm, and were maintained in CaCl_2_ to prevent aging of the hydrogels.

The mechanical properties of the hydrogels were evaluated by measuring stress-strain curves via uniaxial compression at the rate of 1 mm min^−1^ until they were completely fractured^63^. Elastic modulus of each sample was calculated from the slope of the stress-strain linear curves^63^ generated by our data. Ultimate tensile strength was determined as the maximum stress before the samples fractured^63^. Strain failure at fracture was determined by dividing the elongation at the moment of rupture by the initial sample length^63^. These properties were calculated and plotted in graphs using *ε* = Δ*L/Lo* for strain and *σ* = *F/Ao* for stress^63^. Samples included functionalized MWCNT-alginate with 1, 3 and 5 mg/ml of MWCNT and alginate gels as controls.

### Scanning Electron Microscopy (SEM)

En-face and cross sectional images of the dried-gels were acquired using SEM following published procedures^57^. For sample preparation, uniform sized gels containing 1, 3 and 5 mg/ml of MWCNT were made and dried at 80 °C in a hot air oven overnight following published procedures^64^. Gels cultured with cells were not oven heated, but dried under a constant flow of argon overnight in a chemical fume hood. Dried samples were sectioned and coated with gold/palladium (2–3 min) in a sputter coater (Gatan Model 682 Precision etching coating system, Pleasantown, California) and visualized using SEM (Hitachi TM-1000 Tabletop Microscope, Tokyo, Japan) at 1000× to 5000× magnification.

### Cell culture

HeLa-GFP cells were a generous gift from Dr. Renato Aguilera at Dept. of Biological Sciences at UTEP^18^. HeLa cells were grown as monolayers in DMEM supplemented with 10% FCS for several passages before seeding atop the gels^18^. Detached cells were seeded onto gel discs that occupied the entire area of wells in a 48-well tissue culture plate at a density of 10,000 cells per well. Samples included MWCNT-alginate gels (containing 1, 3 and 5 mg/ml of MWCNT/gel) and alginate only along with empty plastic wells (controls). All gels were exposed to UV for 15 min. prior to cell culture for sterilization. Each well with gel was initially incubated with a minimal volume (500 μL) of culture media to facilitate cell attachment. The following day, the volume of the medium was increased to 1000 μL per well and maintained throughout the entire culture period. The seeded cells were then incubated (37 °C, 5% CO_2_) for a period of up to 72 h. The gels were inverted and imaged using an inverted confocal fluorescence microscope (ZEISS LSM 700 confocal, Germany), at various time points following which they were dried overnight for SEM imaging as described earlier. Cell proliferation was estimated as described in the following section.

### Cell adhesion, migration and proliferation

The gels were cut into discs and cultured as described earlier with HeLa. During the culture process, cell adhesion, migration and cluster formation were observed with photomicrography using an inverted biological microscope after 24- and 48-h of culture atop the gels. Samples included MWCNT-alginates 1-, 3-, 5 mg/ml along with alginate and empty plastic wells as controls. For cell counting, gels incubated with cells were carefully transferred to fresh wells, gently rinsed with PBS, overlaid with 1 mL of 0.25% trypsin-EDTA per well and incubated at 37 °C for 10 min. on an orbital shaker (30 rpm). Extracted cells from the first-trypsinization cycle were pelleted by centrifugation, pooled with other cells that were removed in a second cycle of trypsinization and counted using a hemocytometer throughout the entire culture period after 24- and 72-h of culture^62^. Normalized cell proliferation was calculated by dividing the viable cell numbers obtained after 72 h by the initial number of cell adhered after 24 h onto that sample set. Mean values obtained by averaging values from at least 3 samples per case were reported. All cell culture experiments were repeated twice (n = 3 for each experiment).

### Statistical analysis

All samples were present in triplicate unless otherwise mentioned. Data are expressed as the mean ± standard deviation. Microsoft Excel Student’s *t*-test was performed to determine if the averages of any two sample datasets compared were significantly different. *p*-values less than 0.05 were considered significant.

## Additional Information

**How to cite this article**: Joddar, B. *et al*. Development of functionalized multi-walled carbon-nanotube-based alginate hydrogels for enabling biomimetic technologies. *Sci. Rep.* **6**, 32456; doi: 10.1038/srep32456 (2016).

## Supplementary Material

Supplementary Information

## Figures and Tables

**Table 1:** Average rupture strain, ultimate tensile strength, modulus of elasticity and pore size of alginate and MWCNT-alginate gels.

Hydrogel Composition	Rupture Strain, *ε*_*r*_	Ultimate Tensile Strength, *σ*_UTS_	Modulus of Elasticity, E	Average Pore Size
	mm/mm	kPa	kPa	μm
Alginate	0.6434 ± 0.04	103.5 ± 1.4	140 ± 6.4	1.01 ± 0.39
1 mg/ml MWCNT	0.492 ± 0.02	79.5 ± 8.5	165.2 ± 10.8	1.98 ± 1.08
3 mg/ml MWCNT	0.405 ± 0.03	62 ± 11	128.9 ± 22	1.96 ± 0.82
5 mg/ml MWCNT	↓	↓	↓	2.22 ± 1.08
