# Supplementary material for: Development of functionalized multi-walled carbon-nanotube-based alginate hydrogels for enabling biomimetic technologies
Source: Sci Rep. 2016 Aug 31;6:32456. doi: 10.1038/srep32456 (PMC5006027; doi:10.1038/srep32456)
Supplement: Supplementary Information [file srep32456-s1.pdf]

Development of functionalized multi-walled carbon-nanotube-based alginate hydrogels for enabling biomimetic technologies

Binata Joddar1,2*, Eduardo Garcia3•, Atzimba Casas4•, Calvin M. Stewart3,
1Department of Metallurgy, Materials Science and Biomedical Engineering, 2Border Biomedical Research Center.
3Department of Mechanical Engineering, 4Department of Biological Sciences,
The University of Texas at El Paso, 500 W University Avenue, El Paso, TX 79968, USA.
***Corresponding Author: bjoddar@utep.edu; Fax: (915)747-8036, Phone: (915)747-8456.**
•Indicates equal contribution.

Supplementary Figures

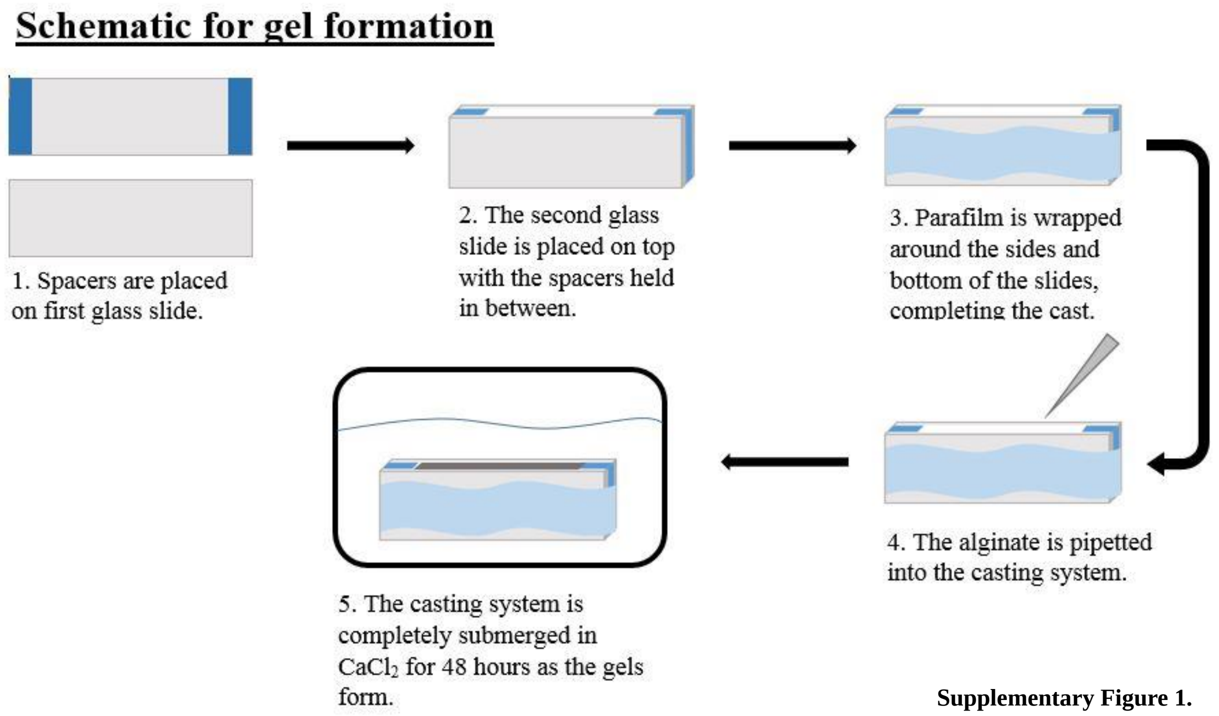

Supplementary Figure 1.

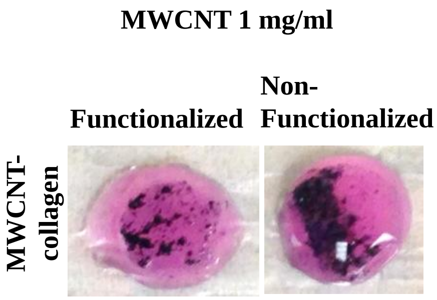

Representative e*n-face* images of MWCNT-collagen (1 mg/ml) gels, functionalized (left) and non-functionalized (right) shown for comparison.

Supplementary Figure 2.

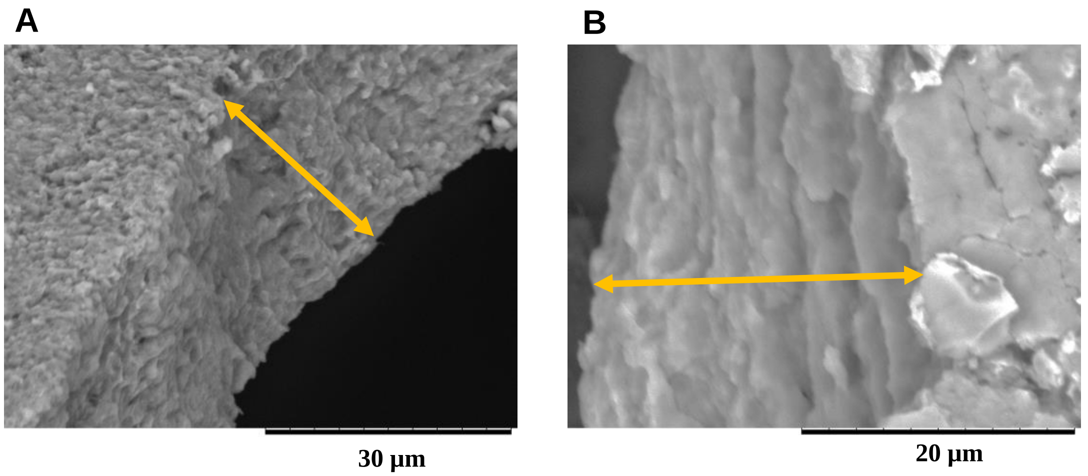

SEM image of the edge of MWCNT-alginate (1 mg/ml) showing laminated sheet-structure of the gels on their edge.

Supplementary Figure 3.

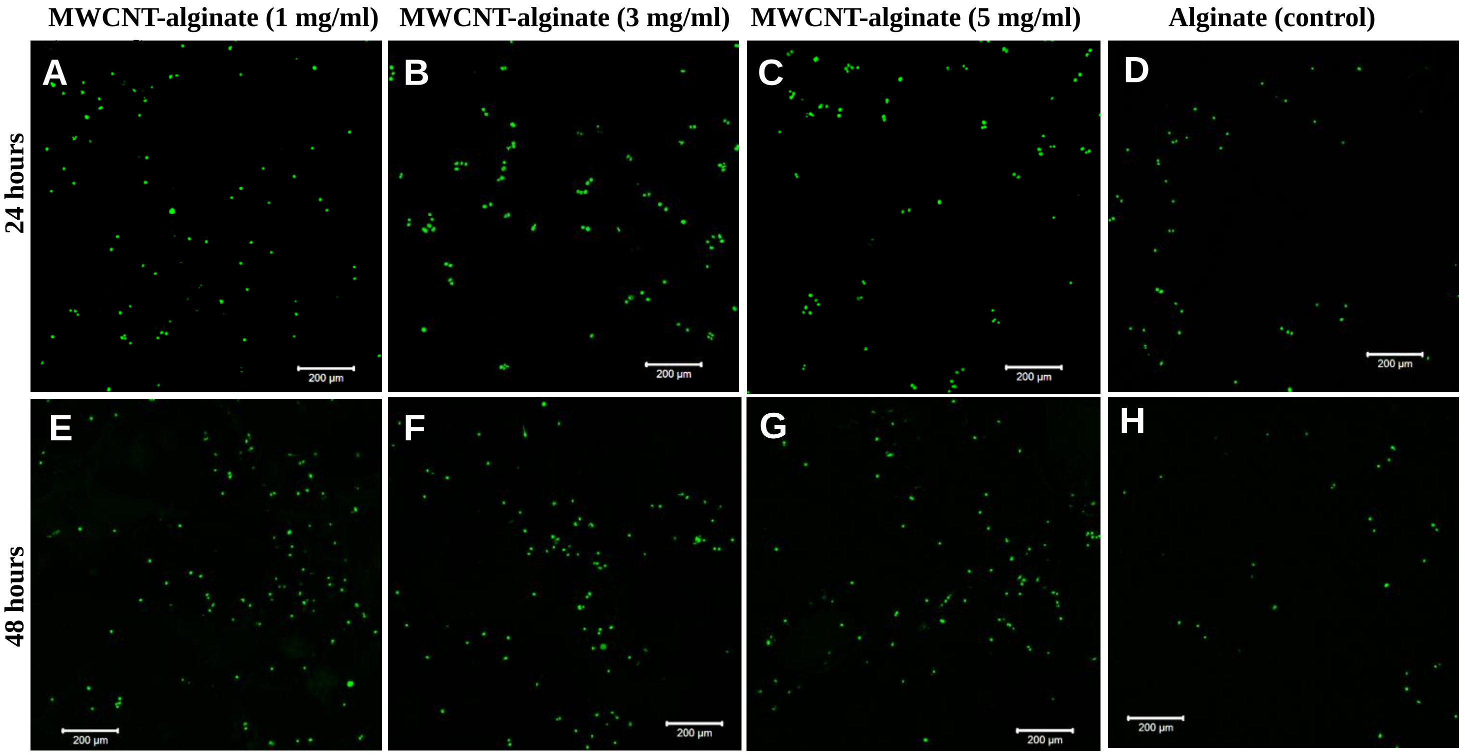

Low magnification images of HeLa-GFP cells cultured on MWCNT-alginate (1, 3, 5 mg/ml) showed agglomeration compared to cells on alginate; in addition, the cells continued to look healthy after 48h.

Supplementary Figure 4.

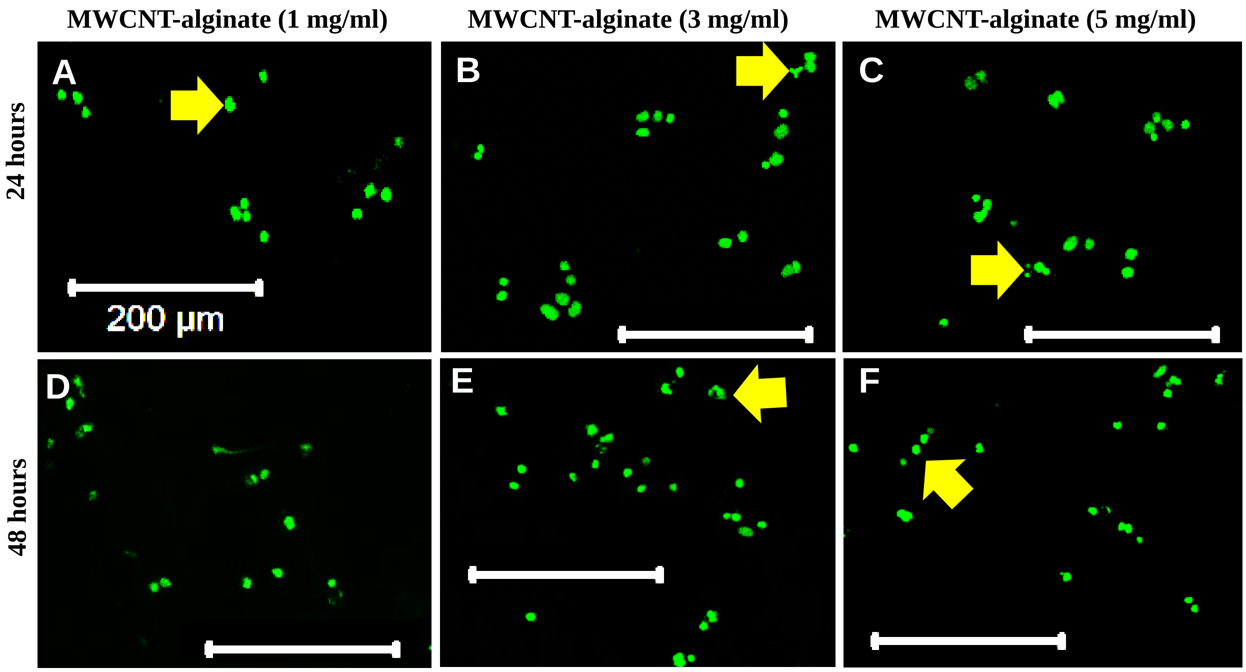

High magnification images of HeLa-GFP cells cultured on MWCNT-alginate (1, 3, 5 mg/ml). Arrows indicate cells actively dividing for proliferation.

Supplementary Figure 5.

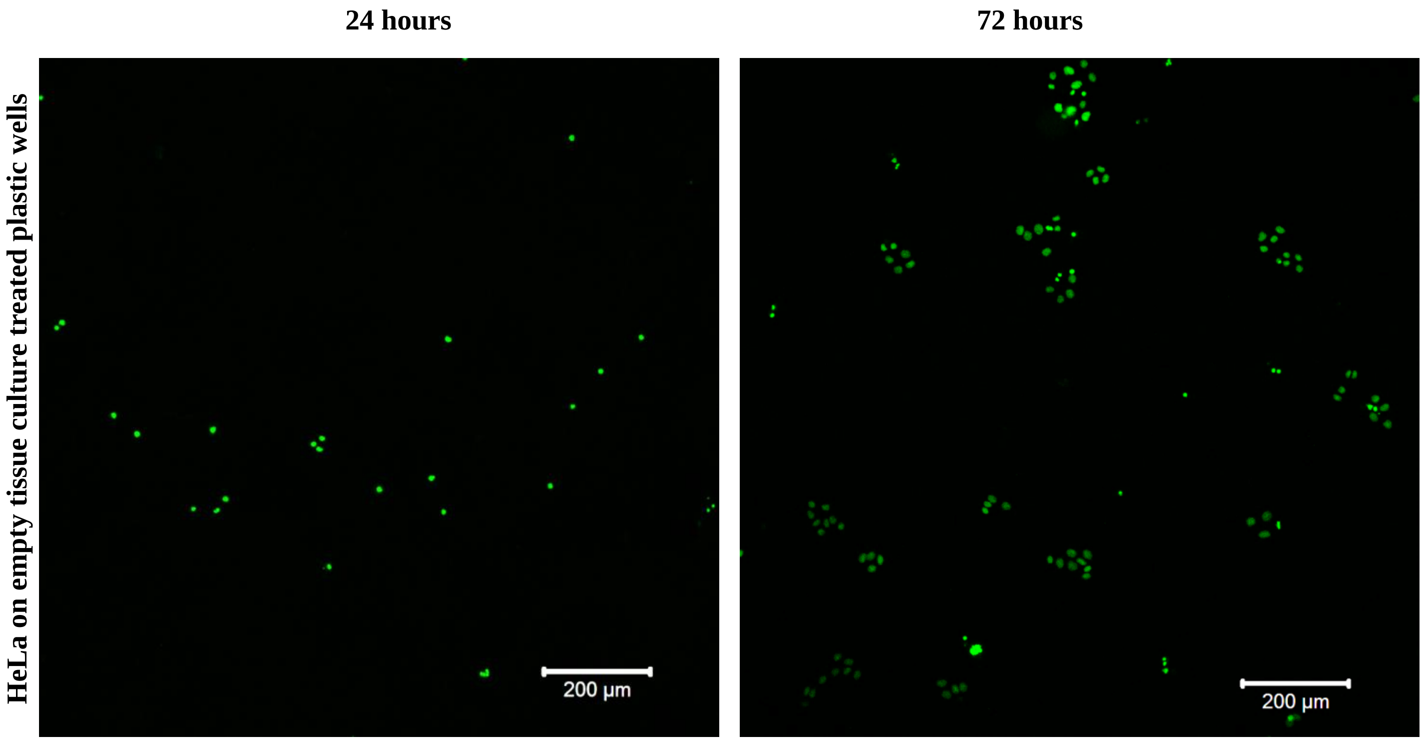

Supplementary Figure 6.
